# Kansuinine A Ameliorates Atherosclerosis and Human Aortic Endothelial Cell Apoptosis by Inhibiting Reactive Oxygen Species Production and Suppressing IKKβ/IκBα/NF-κB Signaling

**DOI:** 10.3390/ijms221910309

**Published:** 2021-09-24

**Authors:** Chen-Sheng Chen, Bo-Yi Pan, Ping-Hsuan Tsai, Fang-Yu Chen, Wen-Chin Yang, Ming-Yi Shen

**Affiliations:** 1 The Ph.D. Program for Cancer Biology and Drug Discovery, China Medical University and Academia Sinica, 91, Hsueh-Shih Rd., Taichung 40402, Taiwan; U105071204@cmu.edu.tw; 2 Graduate Institute of Biomedical Sciences, China Medical University, 91, Hsueh-Shih Rd., Taichung 40402, Taiwan; U106010831@cmu.edu.tw (B.-Y.P.); U105010312@cmu.edu.tw (P.-H.T.); fyc0321@gmail.com (F.-Y.C.); 3 Agricultural Biotechnology Research Center, Academia Sinica, 128, Sec. 2, Academia Rd., Nankang, Taipei 11529, Taiwan; wcyang@gate.sinica.edu.tw; 4 Department of Medical Research, China Medical University Hospital, 91, Hsueh-Shih Rd., Taichung 40402, Taiwan; 5 Department of Nursing, Asia University, 500, Lioufeng Rd., Wufeng, Taichung 41354, Taiwan

**Keywords:** Kansuinine A, anti-atherogenic drug, atherosclerosis, reactive oxygen species, human aortic endothelial cells

## Abstract

Reactive oxygen species (ROS)-induced vascular endothelial cell apoptosis is strongly associated with atherosclerosis progression. Herein, we aimed to examine whether Kansuinine A (KA), extracted from *Euphorbia kansui* L., prevents atherosclerosis development in a mouse model and inhibits cell apoptosis through oxidative stress reduction. Atherosclerosis development was analyzed in apolipoprotein E-deficient (*ApoE*^−/−^) mice fed a high-fat diet (HFD) using Oil Red O staining and H&E staining. Human aortic endothelial cells (HAECs) were treated with KA, followed by hydrogen peroxide (H_2_O_2_), to investigate the KA-mediated inhibition of ROS-induced oxidative stress and cell apoptosis. Oil Red O staining and H&E staining showed that atherosclerotic lesion size was significantly smaller in the aortic arch of *ApoE*^−/−^ mice in the HFD+KA group than that in the aortic arch of those in the HFD group. Further, KA (0.1–1.0 μM) blocked the H_2_O_2_-induced death of HAECs and ROS generation. The H_2_O_2_-mediated upregulation of phosphorylated IKKβ, phosphorylated IκBα, and phosphorylated NF-κB was suppressed by KA. KA also reduced the Bax/Bcl-2 ratio and cleaved caspase-3 expression, preventing H_2_O_2_-induced vascular endothelial cell apoptosis. Our results indicate that KA may protect against ROS-induced endothelial cell apoptosis and has considerable clinical potential in the prevention of atherosclerosis and cardiovascular diseases.

## 1. Introduction

Cardiovascular diseases (CVDs), such as atherosclerosis-mediated myocardial infarction or stroke, are the leading cause of morbidity and mortality. These diseases are responsible for an estimated 17.9 million deaths each year, accounting for 31% of all deaths worldwide and placing a huge economic burden on health care systems globally [1,2]. The progression of CVDs is influenced by several factors, such as aging, hypertension, and atherosclerosis [3]. These factors are associated with a long-term increase in oxidative stress, which causes vascular endothelial damage, due to the overproduction of reactive oxygen species (ROS) [4]. Hydrogen peroxide (H_2_O_2_) is an ROS that induces ischemia-reperfusion injury in animal models, and in vitro studies on CVDs have shown that H_2_O_2_-induced oxidative stress in endothelial cells causes apoptosis [5]. Large amounts of ROS lead to an increase in free radicals and lipid peroxides, which play a role in the pathogenesis of degenerative diseases, such as atherosclerosis [6]. Thus, interventions that inhibit ROS-induced endothelial cell damage would prevent atherosclerosis and provide insights to develop a platform for screening anti-atherogenic drugs to prevent the development of CVDs.

**Figure 1:**
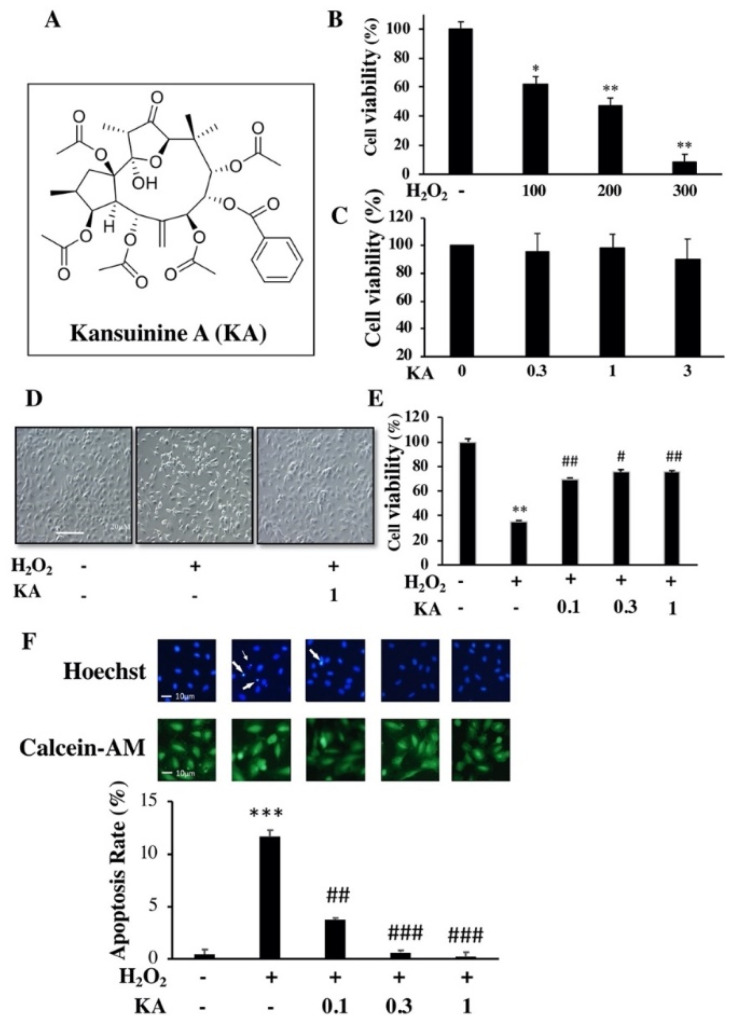
Effects of Kansuinine A (KA) on cell viability and apoptosis. (**A**) The structure of KA. (**B**) Effect of hydrogen peroxide (H_2_O_2_) on the viability of human aortic endothelial cells (HAECs). HAECs were treated with various concentrations of H_2_O_2_ for 24 h. (**C**) Nontoxicity of KA. HAECs were treated with various KA concentrations for 24 h. (**D**) Effect of KA on H_2_O_2_-treated HAECs. Representative image of the effect of KA (1 μM) on the morphology of H_2_O_2_-treated HAECs obtained using an inverted phase-contrast microscope. (**E**) Effect of KA on HAECs damaged by H_2_O_2_. (**F**) Representative images and quantification of Hoechst 33342 (nuclear morphology) and calcein-AM (membrane integrity) staining. HAECs were pre-treated with various KA concentrations (0.1, 0.3, and 1.0 μM) for 1 h, and then with H_2_O_2_ (200 μM) for 24 h. Thereafter, cell viability was determined using the MTT assay. Data are presented as the mean ± standard deviation (n = 3). *p* Values were determined using the Student’s *t*-test. * *p* < 0.05, ** *p* < 0.01, *** *p* < 0.001 vs. control group; # *p* < 0.05, ## *p* < 0.01, ### *p* < 0.001 vs. H_2_O_2_ group.

Medicinal herbs have been widely used to treat various diseases in several countries [7,8,9,10]. To reduce the severity of various diseases, chemicals from plants can be used as complementary and alternative medicinal agents, providing antioxidative, anti-inflammatory, and cell-/tissue-protective effects [11,12], especially for the treatment of CVDs [13,14]. Evidence strongly indicates that several natural products or herbs have potent anti-inflammatory and antioxidant properties [12,14,15]. Kansuinine A (KA; Figure 1A) is extracted from *Euphorbia kansui* L., a well-known medicinal plant in China. *Euphorbia kansui* extract acts on the intestinal smooth muscles to stimulate the intestines, resulting in the propulsion of feces [16]. Furthermore, 3-*O*-(2,3-dimethylbutanoyl)-13-*O*-decanoylingenol, purified from *E. kansui* extract, displays therapeutic potential against allergic diseases, and the underlying mechanism involves the inhibition of intracellular signaling pathways to activate and release chemical mediators from mast cells [17]. Mice treated with stir-fried Radix Kansui and vinegar showed a significant increase in Bcl-2 expression and decrease in caspase-3, NF-κB, and ICAM-1 expression [18]. It is worth noting that KA may reduce inflammation, regulate anti-apoptotic and pro-apoptotic mediators in the mitochondrial pathway [18], and improve the inflammatory response [19].

This study aimed to investigate the effects of KA on H_2_O_2_-induced endothelial cell injury and atherosclerosis development in a mouse model. Additionally, we systematically explored the mechanisms underlying these effects.

## 2. Results

### 2.1. Effects of KA on the Viability of H_2_O_2_-Injured Human Aortic Endothelial Cells (HAECs)

To assess whether KA protects endothelial cells against ROS-induced injury, we first used the MTT assay to examine the viability of H_2_O_2_-injured HAECs. The data showed that H_2_O_2_ reduced HAEC activity in a concentration-dependent manner, and the half maximal inhibitory concentration of H_2_O_2_ was 200 μM (Figure 1B). Additionally, KA at concentrations up to 3 μM did not exert any significant cytotoxic effect (*p* = 0.62, n = 3; Figure 1C); therefore, all subsequent experiments were performed at a dose not higher than 3 μM. The number of cells in the H_2_O_2_ (200 μM) group was significantly reduced compared to that in the control group, and the number of cells in the H_2_O_2_ (200 μM) group that received the KA (1 μM) treatment did not change significantly (Figure 1D). Moreover, the cells were pre-incubated with various KA concentrations (0.1–1.0 μM) for 60 min and then challenged with H_2_O_2_ (200 μM) for 24 h. The results showed that H_2_O_2_ significantly reduced the viability of these HAECs (*p* < 0.01, n = 3). Conversely, KA pre-incubation (0.1–1.0 μM) protected cells from H_2_O_2_-induced cell damage (*p* < 0.01 at 0.1 and 1.0 μM and *p* < 0.05 at 0.3 μM; n = 3; Figure 1E). To further evaluate whether KA can protect HAECs against H_2_O_2_-induced cell damage, HAECs were treated with H_2_O_2_, followed by the addition of KA at various concentrations. After H_2_O_2_ treatment, Hoechst 33342 staining revealed chromosomal breakage fragments, while calcein-AM staining revealed ruptured cell membranes. The results of fluorescence microscopy showed that H_2_O_2_ induced cell apoptosis (Figure 1F; *p* < 0.001 compared with controls). However, the addition of KA reduced the number of chromosomal breakage fragments and maintained cell membrane integrity (*p* < 0.001 at 0.3 and 1.0 μM compared with H_2_O_2_ alone; n = 3; Figure 1F). These results indicate that KA may protect cells from H_2_O_2_-induced damage.

### 2.2. Effect of KA on Intracellular ROS Generation in H_2_O_2_-Treated HAECs

**Figure 2:**
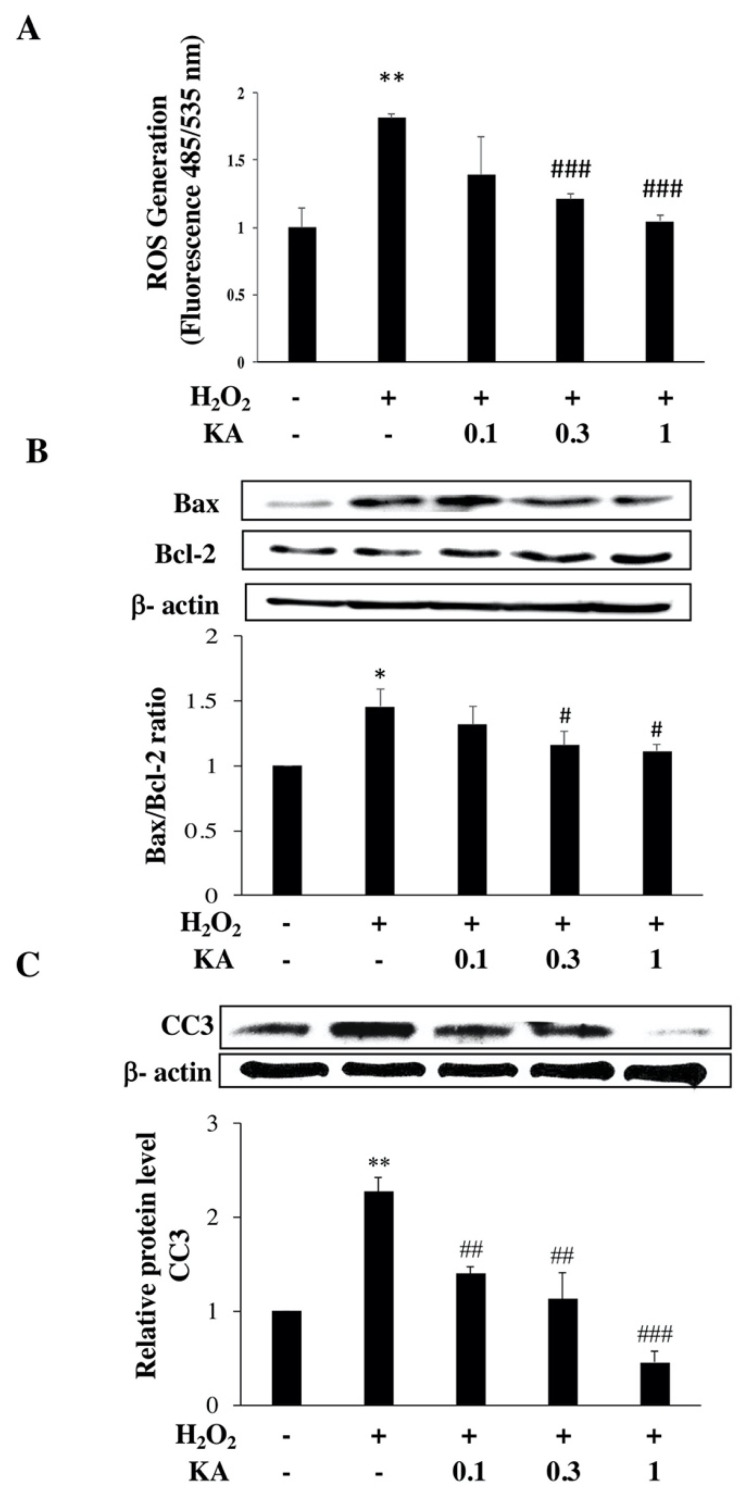
Effects of Kansuinine A (KA) on hydrogen peroxide (H_2_O_2_)-induced reactive oxygen species (ROS) generation, the Bax/Bcl-2 ratio, and cleaved caspase-3 (CC3) protein expression in human aortic endothelial cells (HAECs). HAECs were exposed to KA (0.1, 0.3, and 1.0 μM) for 1 h before H_2_O_2_ (200 μM) treatment for 24 h. (**A**) The cellular ROS/superoxide detection assay was performed. (**B**) Bax/Bcl-2 ratio. (**C**) CC3 expression was evaluated using western blotting. β-actin was used as an internal control. Data are presented as means ± standard deviation (n = 3). *p* Values were determined using the Student’s *t*-test. * *p* < 0.05, ** *p* < 0.01 vs. control group; # *p* < 0.05, ## *p* < 0.01, ### *p* < 0.001 vs. H_2_O_2_ group CC3, Cleaved caspase-3.

To elucidate whether the protective effect of KA is mediated by a reduction in intracellular oxidative stress, intracellular ROS generation was determined using 2′,7′-Dichlorofluorescein diacetate (DCFH-DA). H_2_O_2_ significantly increased the level of ROS, reaching the highest level after 2 h, compared with that in the control group, and KA (0.1–1.0 μM) inhibited this effect in a concentration-dependent manner (Figure 2A). This finding suggests that KA may arrest H_2_O_2_-induced intracellular ROS generation.

### 2.3. Effect of KA on the Protein Expression Levels of Bax, Bcl-2, and Cleaved Caspase-3 (CC3) in H_2_O_2_-Treated HAECs

Expression levels of Bax (pro-apoptotic factor), Bcl-2 (anti-apoptotic factor), and cleaved caspase-3 (CC3, apoptosis marker) in H_2_O_2_-treated HAECs were evaluated using western blotting. The results are expressed as a Bax/Bcl-2 ratio. H_2_O_2_ treatment considerably increased the Bax/Bcl-2 ratio, whereas KA (0.3 and 1.0 μM) significantly reduced this H_2_O_2_-induced effect (Figure 2B). Similarly, the expression of CC3 considerably increased, whereas KA (1 μM) significantly reversed this effect (*p* < 0.001 compared with H_2_O_2_ alone; n = 3) (Figure 2C).

### 2.4. KA Inhibits H_2_O_2_-Induced HAEC Apoptosis via P-IKKβ, P-IκBα, P-NF-κB, and CC3

**Figure 3:**
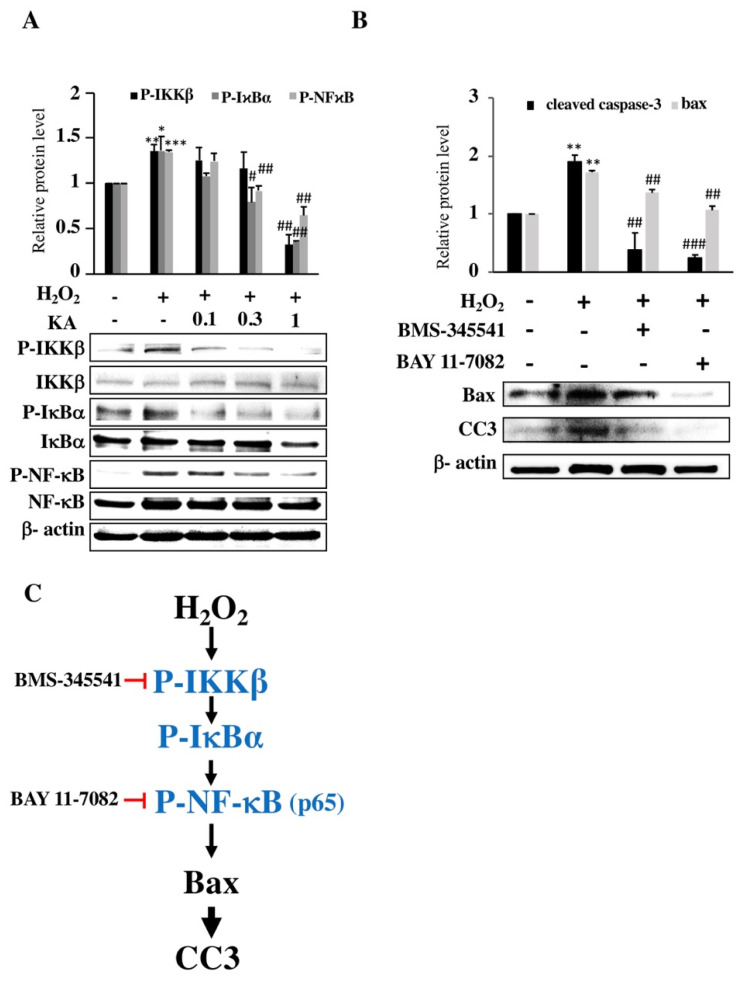
Mechanism of action of hydrogen peroxide (H_2_O_2_) and Kansuinine A (KA). (**A**) Activation of P-IKKβ, P-IκBα, and P-NF-κB (p65). Human aortic endothelial cells (HAECs) were pre-treated with KA (0.1, 0.3, and 1.0 μM) for 1 h and were then exposed to H_2_O_2_ (200 μM) for 24 h. Subsequently, western blotting was performed. (**B**) The expression of Bax and CC3 in the presence or absence of BMS-345541 (25 μM) and BAY 11-7082 (1 μM). Data were quantified by determining the optical density of the bands. The bar graphs show the expression ratio of P-IKKβ, P-IκBα, P-NF-κB(p65), Bax, and CC3 relative to that of β-actin, which was normalized to 1. Data are presented as means ± standard deviation, n = 3, *p* values were determined using the Student’s *t*-test. * *p* < 0.05, ** *p* < 0.01, *** *p* < 0.001 vs. control group; # *p* < 0.05, ## *p* < 0.01, ### *p* < 0.001 vs. H_2_O_2_ (200 μM) group. (**C**) Working model of H_2_O_2_-induced apoptosis. Black arrow = stimulation; red line with end bar = inhibition.

We investigated the molecular mechanism to better understand how KA prevents H_2_O_2_-induced cell apoptosis. Expression levels of P-IKKβ, P-IκBα, P-NF-κB (p65), and CC3 in H_2_O_2_-treated HAECs were evaluated using western blotting. H_2_O_2_ treatment considerably increased expression levels of P-IκBα, P-IKKβ, and P-NF-κB compared with controls (*p* < 0.05, *p* < 0.01, and *p* < 0.001, respectively; n = 3). Conversely, KA significantly reduced the expression of P-IKKβ (*p* < 0.01 at 1.0 µM KA), P-IκBα (*p* < 0.05 at 0.3 µM KA and *p* < 0.01 at 1.0 µM KA), and P-NF-κB (*p* < 0.05 at 0.3 µM and 1.0 µM KA) compared with H_2_O_2_ exposure alone (n = 3; Figure 3A). The H_2_O_2_-induced HAECs were also pre-treated with BMS-345541 (IKK inhibitor, 25 μM) or BAY 11-7082 (NF-κB inhibitor, 1.0 μM), and we found that H_2_O_2_ caused a significant increase in CC3 expression, which was completely reversed by BMS-345541 and BAY 11-7082 (n = 3; Figure 3B). These findings suggest that H_2_O_2_ significantly increased CC3 expression via the IKKβ/IκBα/NF-κB pathway in HAECs (Figure 3C), and this effect was reversed by KA.

### 2.5. KA Reduces the Formation of Atherosclerotic Lesions and the Levels of Apoptosis-Related Proteins in ApoE^−/−^ Mice

**Figure 4:**
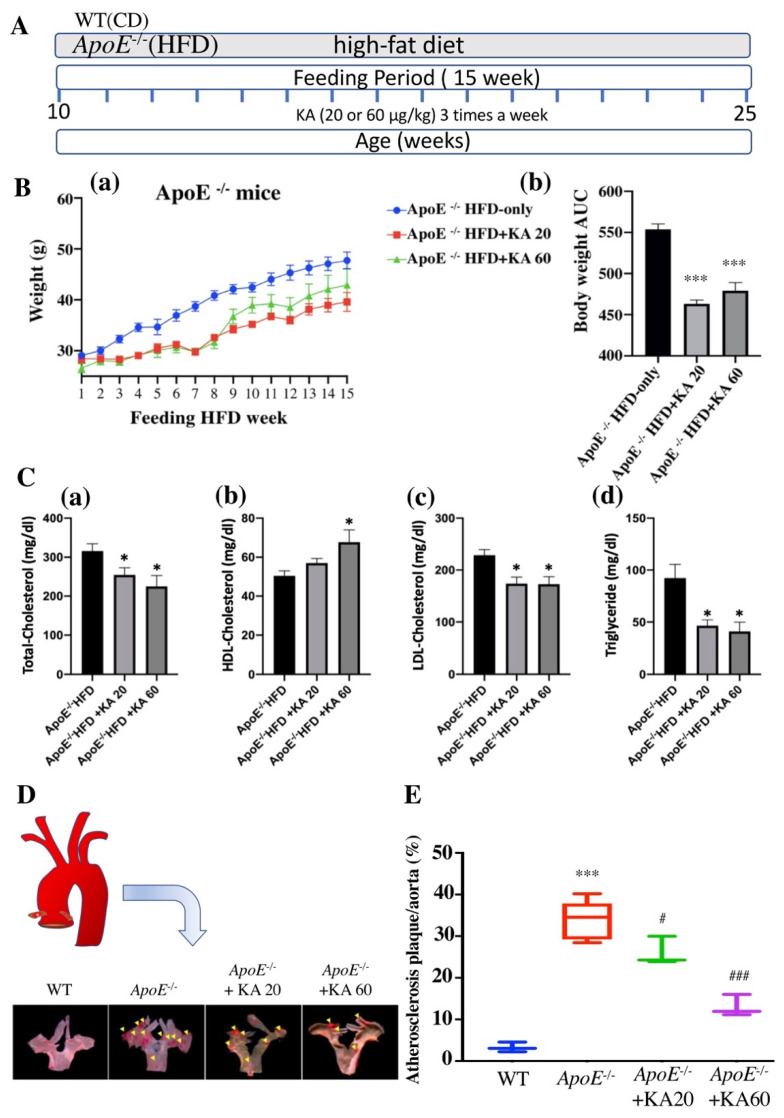
Kansuinine A (KA) reduces atherosclerosis development in *ApoE^−/−^* mice. (**A**) Work scheme. Briefly, C57BL/6 (WT) mice received a normal chow diet (CD) or *ApoE^−/−^* mice received a high-fat diet (HFD) or *ApoE^−/−^* mice received an HFD supplemented with 20 or 60 μg/kg KA per g of body weight three times a week for 15 weeks. (**B**) Mouse body weight was recorded during the 15 week study period. Body weight curves (**a**) and Area Under the Curve values for body weight (g) (**b**). *ApoE^−/−^* mice were fed with a high-fat diet (HFD) or maintained on HFD supplemented with 20 or 60 μg/kg of KA per g of body weight, three times a week for 15 weeks. Area Under the Curve values for the body weight were monitored each week. All data are presented as the means ± SD; statistical analyses were performed using analysis of variance (one-way ANOVA or the Student’s *t*-test), n = 5; *** *p* < 0.001 vs. HFD group. (**C**) Lipid profiles measured at the end of the study period. The plasma lipid profile in *ApoE^−/−^* mice fed an HFD or those supplemented with 20 or 60 μg/kg KA per g of body weight, three times a week for 15 weeks. (**a**) Total-cholesterol (TC), (**b**) HDL-cholesterol, (**c**) LDL-cholesterol, and (**d**) Triglyceride (TG) levels. All data are presented as the means ± SD; statistical analyses were performed using analysis of variance, n = 5; * *p* < 0.05, vs. HFD group. (**D**) Representative aortas from mice in each group stained with Oil Red O. (**E**) En face lesion areas (%) in the aortic arches of wild-type (WT) and *ApoE^−/−^* mice treated with or without KA. The en face luminal aortic surface in WT mice and *ApoE^−/−^* mice fed the HFD, or HFD supplemented with 20 or 60 μg/kg KA (20 KA+HFD and 60 KA+HFD) for 15 weeks (n = 5 per group). Data represent the mean ratio of plaque area over the total aortic luminal area and are expressed as means ± standard deviation. *p* Values were determined using one-way analysis of variance (ANOVA) followed by Scheffé’s method. *** *p* < 0.001 vs. WT group; # *p* < 0.05, ### *p* < 0.001 vs. HFD group.

**Figure 5:**
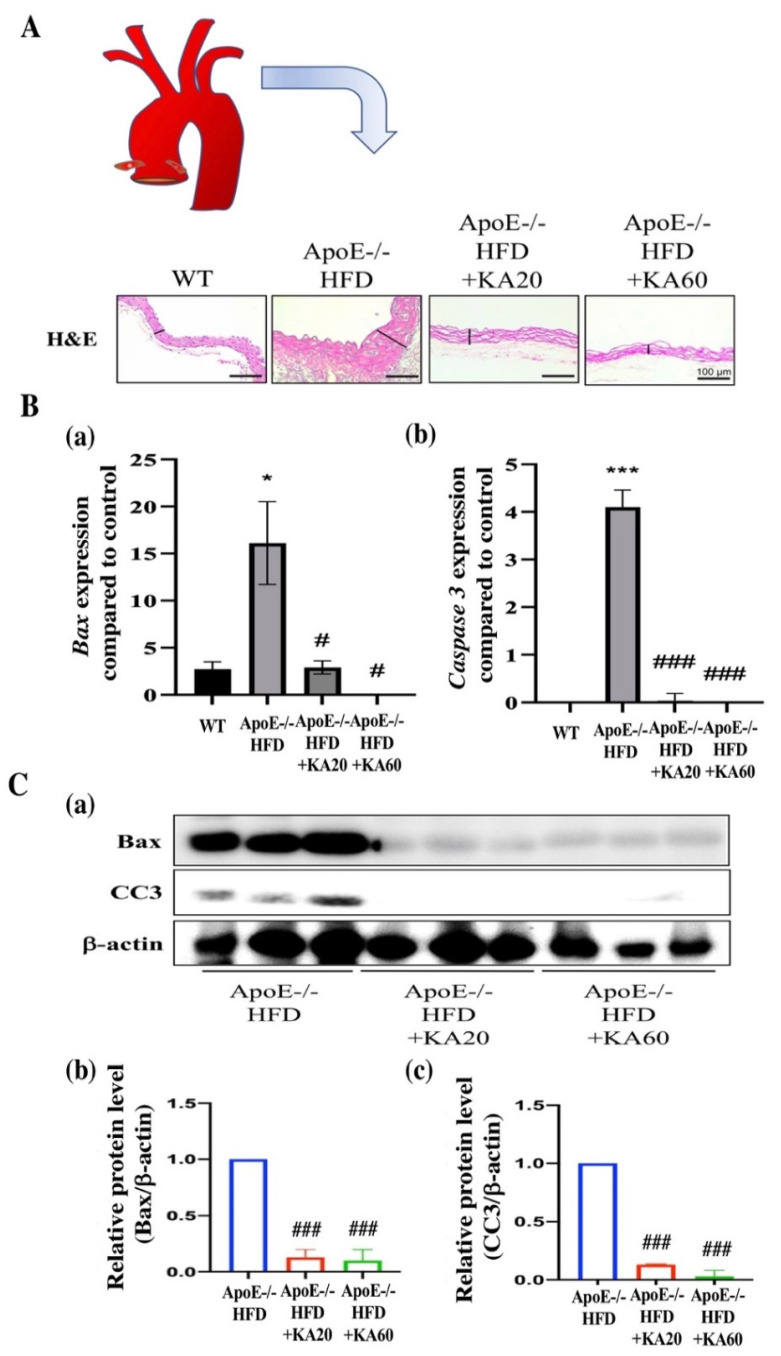
Kansuinine A (KA) reduces atherosclerotic lesions. Results showing the mRNA and protein expression of Bax and caspase-3 in *ApoE^−/−^* mice. *ApoE^−/−^* mice fed a high-fat diet (HFD) or HFD supplemented with 20 or 60 μg/kg KA (20 KA+HFD and 60 KA+HFD, respectively) per g of body weight three times a week for 15 weeks. (**A**) Hematoxylin and eosin (H&E) staining of representative atherosclerotic lesions in aortic root from WT and *ApoE^−/−^* mice. Lesion width of aortic root highlighted with black. Scale bars, 100 µm. (**B**) mRNA expression of Bax (**a**) and caspase-3 (**b**) were evaluated using RT-qPCR. (**C**) Protein expression of Bax (**b**) and cleaved caspase-3 (CC3, **c**) were evaluated using western blotting (**a**). β-actin was used as the control. Data are presented as means ± standard deviation (n = 3). *p* Values were determined using the Student’s *t*-test. * *p* < 0.05, *** *p* < 0.001 vs. WT mice; # *p* < 0.05, ### *p* < 0.001 vs. *ApoE^−/−^* mice fed a high-fat diet (HFD group).

ROS-induced vascular endothelial cell damage is an essential risk factor for the pathogenesis of atherosclerosis [4,20,21]. To further study the effects of KA on the development of atherosclerotic lesions in *ApoE*^−/−^ mice, the most popular murine model used for atherosclerotic studies, the bodyweight of the *ApoE*^−/−^ mice treated with 20 or 60 μg/kg KA was found to be significantly lower compared with that of mice in the HFD group (Figure 4A,B; *p* < 0.001). The serum total-cholesterol, low-density lipoprotein (LDL)-cholesterol, and triglyceride levels of 20 and 60 μg/kg KA-treated groups were significantly lower in *ApoE*^−/−^ mice than in HFD mice (*p* < 0.05), while the HDL-C levels of the 60 μg/kg KA-treated group were significantly higher in *ApoE*^−/−^ mice than in HFD mice (Figure 4C). These results demonstrate that KA effectively ameliorates the serum biochemical parameters. Moreover, we used Oil Red O staining to examine lesions in the aortas of *ApoE*^−/−^ mice. After the administration of KA, the aortic arch assay was performed to evaluate lesion formation. In *ApoE*^−/−^ mice fed the HFD for 15 weeks, the atherosclerotic lesion was larger than that in wild-type (WT) mice (*p* < 0.001; Figure 4C). However, the atherosclerotic lesion area was considerably lower in HFD-fed *ApoE*^−/−^ mice supplemented with 20 μg/kg KA (23% reduction, *p* < 0.05) and 60 μg/kg KA (61% reduction, *p* < 0.001) (Figure 4D,E) than in HFD-fed mice not supplemented with KA. In addition, we also used hematoxylin and eosin (H&E) staining for cross-sections of the aorta. *ApoE*^−/−^ mice administered KA demonstrated a significant attenuation of aortic plaque formation in comparison with untreated mice. The plaque area was reduced by KA treatment in a dose-dependent manner (Figure 5A). At the 60 μg/kg dose, KA almost completely abrogated plaque formation in comparison with untreated *ApoE*^−/−^ mice. Furthermore, we found that mRNA expression of *Bax* and *caspase 3* and protein expression of Bax and CC3 were reduced by KA treatment in aortic tissues (Figure 5B,C). Together, these results suggest that KA attenuated the size of atherosclerotic lesions.

### 2.6. KA Modulates the Expression Levels of Glutathione Peroxidase (GPx) and Malondialdehyde (MDA)

**Figure 6:**
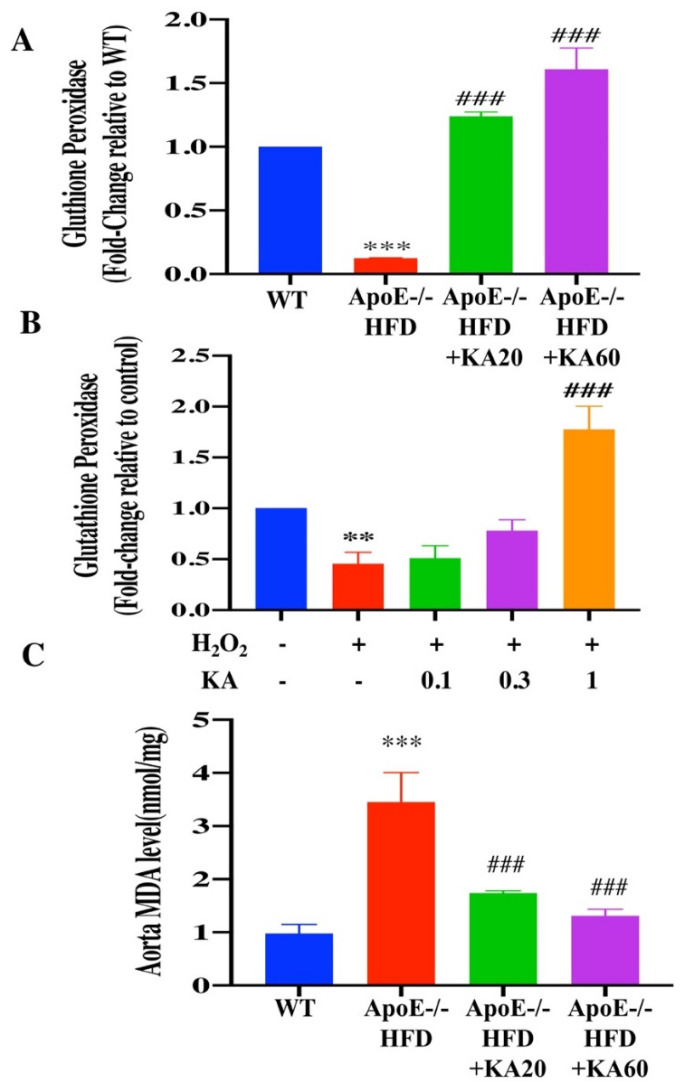
Effects of Kansuinine A (KA) on glutathione peroxidase (GPx) activity in vivo or in vitro and the aorta MDA level in mice. Activity of GPx was measured in (**A**) aortic tissue homogenates from mice in the different groups and (**B**) human aortic endothelial cells exposed to KA (0.1, 0.3, and 1.0 μM) for 1 h before H_2_O_2_ (200 μM) treatment for 24 h. Data are presented as the means ± standard deviations (n = 5). *p* Values were determined using the Student’s *t*-test. ** *p* < 0.01, *** *p* < 0.001 vs. WT or control group; ### *p* < 0.001 vs. *ApoE*^−/−^ mice fed a high-fat diet (HFD) or cells from the H_2_O_2_ group. (**C**) MDA levels in aorta of mice. *ApoE*^−/−^ mice fed an HFD or maintained on an HFD supplemented with 20 or 60 μg/kg KA per g of body weight three times a week for 15 weeks. Data are presented as the means ± standard deviations (n = 5). *p* Values were determined using the Student’s *t*-test. *** *p* < 0.001 vs. *ApoE*^−/−^ mice fed an HFD. ### *p* < 0.001 vs. *ApoE*^−/−^ mice fed an HFD.

Oxidative stress and oxidative tissue injury markers were evaluated to determine whether KA protects against oxidative cell damage. Levels of the antioxidant enzyme glutathione peroxidase (GPx) were significantly reduced in the aorta of *ApoE*^−/−^ HFD-fed mice, compared with levels observed in WT mice (Figure 6A; *p* < 0.001). *ApoE*^−/−^ mice fed an HFD, along with KA20 or KA60, showed significantly higher levels of GPx than mice from the *ApoE*^−/−^ HFD group (Figure 6A; *p* < 0.001). Moreover, in vitro, H_2_O_2_ treatment decreased the levels of GPx (fold-change relative to control) (Figure 6B; *p* < 0.01) in HAECs. Conversely, GPx levels increased significantly upon treatment with KA (Figure 6B; *p* < 0.001 at 1.0 µM KA). Moreover, levels of the lipid peroxidation marker malondialdehyde (MDA) were significantly elevated in the *ApoE*^−/−^ HFD group, compared with that in the WT group (Figure 6C; *p* < 0.001). MDA levels in *ApoE*^−/−^ mice fed an HFD, along with KA20 or KA60, were significantly lower than those in mice from the *ApoE*^−/−^ HFD group (Figure 6C; *p* < 0.001).

## 3. Discussion

**Figure 7:**
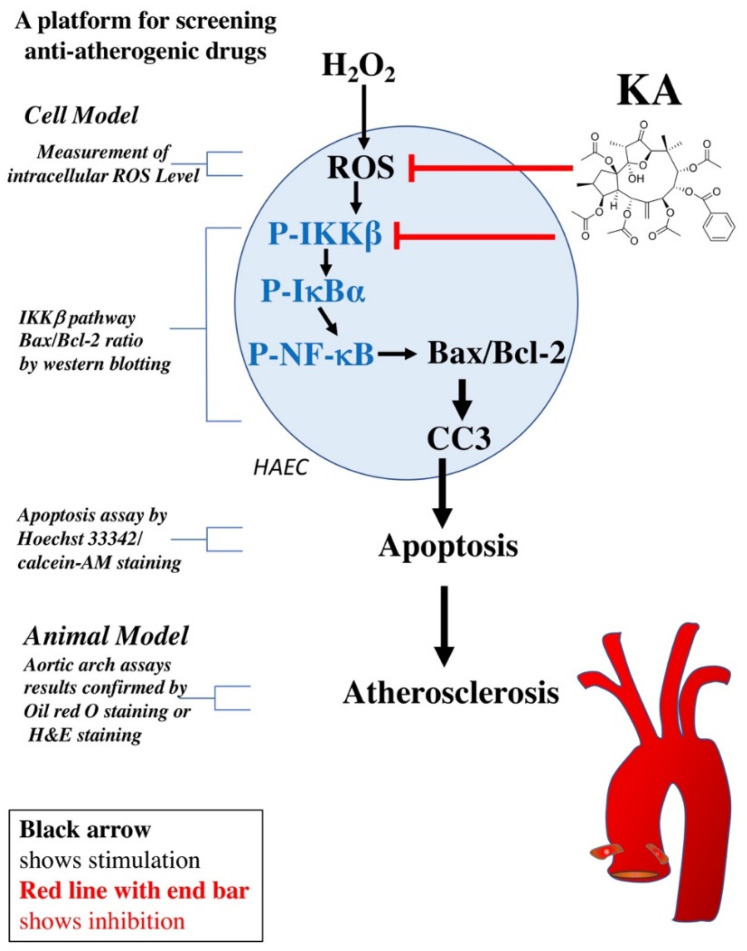
A platform for screening anti-atherogenic drugs and Kansuinine A against hydrogen peroxide-mediated cell apoptosis and atherosclerosis. The black arrow indicates stimulation, and the red line with an end bar indicates inhibition.

It is well known that endothelial dysfunction caused by ROS is one of the major mechanisms of CVD development [21]. The inhibition of ROS-induced endothelial cell damage is beneficial in preventing CVDs and atherosclerosis. Therefore, an approach (combining the measurement of intracellular ROS levels, western blotting, apoptotic cell staining, and aortic arch assays) was developed to screen anti-atherogenic drugs. Based on the screening platform, the findings of the present study indicate that KA protects HAECs from ROS-induced cellular damage. Moreover, this effect may be mediated by a decreased Bax/Bcl-2 ratio and phosphorylation of IKKβ, IκBα, NF-κB, and CC3. In addition, the findings showed that atherogenic changes in the aortas of *ApoE*^−/−^ mice can be prevented by KA (Figure 7).

Hydrogen peroxide, an ROS, has been shown to play a major role in vascular and endothelial dysfunction [20]. Our findings corroborate those of previous studies indicating that H_2_O_2_ may produce intracellular ROS and inhibit cell viability, depending on its concentration [22]. Due to the importance of inflammation and oxidation pathways in atherosclerosis, nutraceuticals may be of great value to help reduce the risk from a macroscopic and pathophysiological perspective [23]. Moreover, herbs have been widely used to treat diseases in several Asian countries [7,9]. There is sufficient evidence that several herbs have significant antioxidant properties [15]. KA is an anthraquinone compound extracted from *E. kansui* with ethyl acetate; our findings showed that KA inhibits H_2_O_2_-induced intracellular ROS generation and that 3 μM KA is not harmful to cells.

There are many enzymes involved in ROS production, including NADPH oxidase (NOX), mitochondrial electron transport chain (ETC), xanthine oxidase, uncoupled endothelial nitric oxide synthase (eNOS), cytochrome P-450 oxygenase, cyclooxygenase, and oxygenase. Vascular NOX subtypes (Nox1, Nox2, Nox4, and Nox5) differ in their activity and cell specificity in response to agonists, growth factors, and hypoxia, and the types of ROS released after activation [24]. Imbalanced ROS homeostasis leads to oxidative stress, leading to direct cell damage, and destruction of the ROS signal transduction mechanism will exacerbate this damage [25]. Moreover, ROS-mediated cell injury occurs mainly via DNA damage and apoptosis [21,26]. Therefore, to assess H_2_O_2_-induced apoptosis and the inhibitory effect of KA on H_2_O_2_, we performed Hoechst fluorescence staining [10]. Under the action of H_2_O_2_, the damaged DNA was stained and produced strong fluorescence. Additionally, calcein-AM live-cell staining can also be used to observe damaged cells that produce strong fluorescence under the action of H_2_O_2_; this effect can be inhibited by KA at various concentrations, and the inhibitory effect is proportional to the dose of KA [27].

Furthermore, KA may inhibit cell apoptosis [28]. Several apoptosis-related proteins regulate cell survival, including Bax, Bcl-2, and CC3 [10,29,30]. H_2_O_2_ causes cell death via the apoptosis pathway, and this can be confirmed by the protein expression of Bax (pro-apoptotic protein) and Bcl-2 (anti-apoptotic protein) and the Bax/Bcl-2 ratio [30]. Experimental results showed that KA reduces the expression of Bax and the Bax/Bcl-2 ratio, eventually decreasing cell apoptosis. In addition, Nrf2 and NF-κB are key pathways closely associated with oxidative stress and inflammation [12]. NF-κB is a central mediator of inflammation, and its signaling is considered to be a master regulator of the inflammatory response and inflammation-related diseases [12,31,32]. Although IKKβ, IκBα, and NF-κB are inflammatory factors, they are also involved in the apoptosis pathway [32,33]. Our results suggest that this may be mediated by reducing the phosphorylation of IKKβ, IκBα, and NF-κB. Moreover, anti-inflammatory drugs can also reduce cell apoptosis caused by ROS [34]. Cell survival regulation requires several apoptosis-related proteins, including IKKβ, IκBα, NF-κB, and CC3. Our experiments verified that KA reduces the expression of these proteins, which enhances the apoptosis pathway following H_2_O_2_ stimulation, and increases the inhibitory effect at increased concentrations. H_2_O_2_ increased the concentration of IKKβ, IκBα, and NF-κB, and the Bax/Bcl-2 ratio, leading to caspase-3 cleavage and apoptosis, whereas KA pre-treatment inhibited H_2_O_2_-induced apoptosis [35,36].

Vascular endothelial cell exposure to ROS leads to cell damage followed by atherosclerosis [4]. Studies have also shown that H_2_O_2_ can cause vasodilation in mice [37]. Recent antioxidant interventions have been established using synthetic and naturally occurring molecules as adjuvant strategies for lipid reduction or anti-inflammatory therapies, aimed at reducing the risk of CVDs [21,26]. In the present study, we further confirmed that KA substantially decreased the size of atherosclerotic lesions. According to the results of several previous studies, malondialdehyde (MDA) is one of the most frequently used indicators of lipid peroxidation. The MDA levels showed a positive correlation with the major CVD risk factors. In our study, it was found that the MDA level in the aorta of *ApoE*^−/−^ HFD-fed mice was significantly higher than that in the aortas of WT mice (Figure 6C; *p* < 0.001). However, MDA levels in *ApoE*^−/−^ mice fed an HFD, along with KA20 or KA60, were significantly lower than those in mice from the *ApoE*^−/−^ HFD group (Figure 6C; *p* < 0.001). Thus, the KA-mediated inhibition of ROS generation in vivo may partially be attributed to KA’s free radical-scavenging activity. In addition, we also attempted to replicate the molecular experiments of KA as an inhibitor of the P-IKKβ/p65/Bax/cleaved caspase-3 (CC3) pathway in *ApoE*^−/−^ mouse tissue and aortic sections. We found that mRNA expression of *Bax* and *caspase 3* and the protein expression of Bax and CC3 were reduced by KA treatment in aortic tissues, as we found in in vitro cell experiments (Figure 5B,C). However, KA decelerates the accumulation of lipid/plaque in mice, and the underlying mechanism has not been fully clarified, because the development of atherosclerosis is complicated. In atherosclerosis, leukocytes and platelets enhance the interaction with endothelial cells by the upregulation of cell adhesion molecules. In addition, the phenotypic vascular smooth muscle cells (VSMCs) undergo enhanced proliferation, migration, and invasion, thereby narrowing the arterial lumen and inducing angiotensin dysregulation [38]. In particular, VSMCs can proliferate and synthesize extracellular matrix and secrete adhesion molecules, thereby recruiting and stabilizing inflammatory cells and absorbing lipids, and promoting the formation of foam cells, leading to the development of atherosclerotic lesions [39]. Hence, atherosclerosis can be described as an immune disease that is activated via the inflammatory pathway. Herbal preparations can suppress pathological immune responses and can regulate the progression of atherosclerosis [40]. A detailed investigation is needed to clarify whether KA can modulate the diameter of blood vessels, regulate blood pressure, and improve blood flow, as well as elucidate the role of immune cells.

In addition to H_2_O_2_-induced ROS, other stimuli (such as high concentrations of glucose and atherogenic LDL) exhibit similar effects [41]. According to the results of existing clinical trials, natural products can be used as adjuvant therapeutics to reduce the level of small dense LDL and the number of LDL particles, increase the size of LDL, and, thereby, prevent CVD [42]. In turn, oxidized LDL can activate abnormal endothelial cells, thereby exposing cell adhesion molecules (VCAM-1 and ICAM-1) that bind and recruit inflammatory leukocytes (T cells and monocytes) to the subendothelial space [43]. Therefore, diseases related to diabetes and dyslipidemia, as well as treatments of these diseases, can also be further investigated using this platform. In addition, because mitochondria are an important source of ROS and contribute to oxidative stress in cells under pathologic conditions, in preliminary studies, we also found that mitochondrial function/structure is impaired in HAECs treated with H_2_O_2_. However, Kansuinine A may improve H_2_O_2_-induced mitochondrial dysfunction.

There are many related enzymes (such as NOX, catalase, and glutathione peroxidase) involved in the production of ROS. With regard to the effects of KA in protecting cells from oxidative stress, as mediated by antioxidant enzymes, we found that the level of GPx (fold-change) in the aortas from the *ApoE*^−/−^ HFD-fed mice was significantly lower than that in the aortas from the WT mice (Figure 6A; *p* < 0.001). Conversely, GPx activity was significantly lower in the *ApoE*^−/−^ mice fed an HFD, along with KA20 or KA60 (Figure 6A; *p* < 0.001). Moreover, H_2_O_2_ treatment considerably decreased GPx levels (fold-change relative to control) (Figure 6B; *p* < 0.01) in HAECs. However, GPx levels increased significantly upon treatment with KA (Figure 6B; *p* < 0.001 at 1.0 µM KA). Furthermore, we verified that KA exerts a protective effect on HAECs by reducing the effect of ROS. Whether KA has the same protective effect in the presence of other stress-inducing molecules, such as reactive oxygen-nitrogen species, hormones, and other drugs, must be assessed further. Future studies are also warranted to explore other mechanisms of KA.

## 4. Materials and Methods

### 4.1. Reagents and Antibodies

KA (HPLC purity >98%; Figure 1A) was purchased from Aobious (Gloucester, MA, USA). H_2_O_2_, dimethyl sulfoxide (DMSO), and BMS-345541 were purchased from Sigma-Aldrich (Merck KGaA, Darmstadt, Germany). Trypan blue and fetal bovine serum were purchased from Corning (Manassas, VA, USA) and Gibco (Auckland, New Zealand), respectively. 3-(4,5-Dimethylthiazol-2-yl)-2,5-diphenyltetrazolium bromide (MTT) and cellular ROS/Superoxide detection assay kit were purchased from Merck KGaA and Abcam (Cambridge, MA, USA), respectively. Anti-Phospho-IKKβ, anti-Phospho-IκBα, anti-Phospho-NF-κB (p65), and anti-cleaved caspase-3 antibodies were obtained from Cell Signaling Technology (Danvers, MA, USA). Anti-β-actin antibodies were obtained from Sigma-Aldrich. BAY 11-7082 was procured from Calbiochem (La Jolla, CA, USA). All other reagents and chemicals were of analytical grade.

### 4.2. HAEC Culture

The HAEC line was isolated from the human ascending and descending aorta (PromoCell GmbH, Heidelberg, Germany). The cells were cultured in an endothelial cell growth medium at 37 °C in a humidified atmosphere of 5% CO_2_. During cell culture, the medium was changed every three days until the cells reached 90% confluence. To assess the effects of KA on HAECs, the cells were treated with increasing concentrations of KA (0.1, 0.3, and 1.0 μM) for 1 h, and then with H_2_O_2_ (200 μM) or vehicle for 24 h unless stated otherwise [10].

### 4.3. Measurement of Cell Viability

The MTT assay was used to determine cell viability. The cells, at a density of 1.0 × 10^5^ cells/well, were seeded in a 96-well plate and allowed to attach for 24 h. Thereafter, the culture supernatant was removed, and the cells were incubated with the MTT solution (5 mg/mL) supplemented with endothelial cell growth medium at 37 °C for 4 h. Subsequently, we replaced the dye-containing medium with 150 μL DMSO and agitated the plate using a plate shaker for 5 min. The optical density of the sample in each well was measured using a microplate reader (Infinite M1000; TECAN, Mechelen, Belgium) at 560 nm. Cell viability is expressed as a percentage relative to the control [28].

### 4.4. Apoptotic Cell Staining

Hoechst 33342 (Molecular Probes, Eugene, OR, USA) and calcein acetoxymethyl ester (calcein-AM, Molecular Probes) staining were used to assess chromosomal breakage and cell membrane integrity, respectively. HAECs were pre-treated with vehicle (0.1% DMSO) or KA (0.1, 0.3, or 1.0 μM) for 30 min and incubated with or without H_2_O_2_ (200 μM) for 24 h. The treated cells were stained with 1.0 μM Hoechst 33342 and calcein-AM for 10 min. An Olympus IX70 inverted microscope (Olympus Corporation, Center Valley, PA, USA) was used for fluorescence imaging, and the apoptotic cells were quantified according to a previously described method [10].

### 4.5. Measurement of Intracellular ROS Levels

Intracellular ROS levels were determined using the Cellular ROS Assay Kit (ab113851) according to the manufacturer’s instructions. Briefly, the cells (1.0 × 10^5^ cells/well) were seeded in a 96-well plate and allowed to attach for 24 h. Thereafter, the culture supernatant was removed, and the cells were washed with 1× assay buffer (100 μL/well). DCFH-DA, an ROS-specific staining agent, was added to the cells, and the plate was incubated for 60 min in the dark. After incubation, the intracellular ROS levels were measured using a fluorescence microplate reader (Infinite M1000; TECAN) (Ex = 488 nm, Em = 520 nm).

### 4.6. Western Blotting

HAECs were culture in six-well plates at a density of 1.0 × 10^6^ cells/well and allowed to attach for 24 h. HAECs were treated with vehicle, only H_2_O_2_ (200 μM; 24 h), or H_2_O_2_ (200 μM; 24 h) and KA (0.1, 0.3, and 1.0 μM; 1 h before adding H_2_O_2_). Thereafter, the HAECs were homogenized and lysed in the presence of a protease inhibitor (Roche Applied Science, Penzberg, Germany) in RIPA buffer (Thermo Fisher Scientific Inc., Waltham, MA, USA) to obtain the extracted protein. The protein sample was separated by sodium dodecyl sulfate-polyacrylamide gel electrophoresis (SDS-PAGE) (110 V, 90 min). The separated proteins were transferred onto a polyvinylidene fluoride (PVDF) membrane and blocked with phosphate-buffered saline Tween-20 (PBST) supplemented with 5% bovine serum albumin for 60 min. Thereafter, the membranes were incubated with specific primary antibodies (1:1000), including Bax (Arigobio, Hsinchu, Taiwan), Bcl-2 (Arigobio), cleaved caspase-3 (CC3) (Cell Signaling Technology), and β-actin (1:10,000; Sigma-Aldrich), in PBST and incubated overnight at 4 °C. The membranes were incubated with anti-rabbit horseradish peroxidase (HRP)-conjugated immunoglobulin G (IgG) (1:1000 in PBST; Gene Tex) for 60 min at 25 °C to detect the binding of its corresponding antibody. Immunoreactive bands were detected with ECL Reagent (Millipore, Billerica, MA, USA). For the quantitative analysis of protein expression, the optical density of protein bands was analyzed by video densitometry (G-box Image System; Syngene, Frederick, MD, USA) [10].

### 4.7. Lipid and Lipoprotein Profile

Mice were fasted for 12–14 h before blood samples were collected. Serum was separated by centrifugation at 3000× *g* for 10 min. Total cholesterol, triglyceride, very-low-density lipoprotein cholesterol, low-density lipoprotein cholesterol, and high-density lipoprotein cholesterol serum levels were enzymatically determined using commercial kits and the SPOTCHEM EZ SP-4430 automated analyzer (ARKRAY, Inc., Kyoto, Japan).

### 4.8. Experiments with Mice and Oil Red O Staining of the Aortas

Experiments with mice were approved by the China Medical University Institutional Animal Care and Use Committee (permit no. CMUIACUC-2019-094) and performed in accordance with the *Guide for Care and Use of Laboratory Animals* published by the U.S. National Institutes of Health (NIH Publication No. 85-23, revised 1996). Two species of mice were used to determine the effects of KA on atherosclerosis progression and divided into four groups (n = 5 per group): (1) mice were maintained on a normal chow diet (CD); (2) apolipoprotein E knockout mice (*ApoE*^−/−^ mice; from Jackson Laboratory, Sacramento, CA, USA) were fed a normal diet and HFD (TestDiet58Y1) for 15 weeks; and (3) and (4) mice were maintained on an HFD supplemented with 20 or 60 μg/kg KA per g of body weight, respectively, three times a week for 15 weeks. KA was administered via intraperitoneal injection. After the 15 week study period, the mice were anaesthetized and sacrificed by cervical dislocation, and the aortic arches were removed. Atherosclerotic plaques were visualized using Oil Red O staining. The aortic arches were fixed with 4% paraformaldehyde for 15 min and stained with 0.5% Oil Red O solution (Sigma-Aldrich) for 24 h [44]. The stained aortas were spread on a black board and photographed using a digital camera under identical light conditions with the same photography parameters. We calculated the area of the plaque divided by the area of the total blood vessels. Pictures were analyzed using Image J (Version 1.52a, NIH, Bethesda, MD, USA).

### 4.9. Histological Investigations

Mice were anesthetized and then euthanized by cervical dislocation. Subsequently, their thoracic aortas were excised. The aortic roots were fixed using 4% paraformaldehyde overnight and embedded in paraffin to obtain serial 3-µm-thick sections. Every third slice in the series was stained using H&E.

### 4.10. RNA Isolation and Quantitative Real-Time PCR

Tissues were snap-frozen in liquid nitrogen and stored at −80 °C until further processing. Total RNA from arteries was isolated using the NucleoZOL reagent (Macherey-Nagel GmbH & Co., KG, Düren, Germany). Reverse transcription was conducted using the iScript gDNAClear cDNA synthesis kit (Bio-Rad, Hercules, CA, USA). Subsequently, quantitative real-time-PCR analyses were performed using the SensiFAST SYBR kit (BIO-98020, BioLin, Eveleigh, Australia) on the StepOnePlus real-time PCR system (Applied Biosystems, Life Technologies, Carlsbad, CA, USA) according to the manufacturer’s instructions. The sequences of the primers used were: *caspase-3*, 5′-GCGAGTGAGAATGTGCATAAATTC-3′ and 5′GGGAAACCAACAGTACTCAGTCCT-3′; *Bax*, 5′-GTTTCATCCA GGATCGAGCAG-3′ and 5′-AGCTGAGCGAGTGTCTCCGGCG-3′; and *GAPDH*, 5′-AGAAGGCTGGGGCTCATTTG-3′ and 5′-AGGGGCCATCCACAGTCTTC-3′. Relative gene expression was calculated using the delta delta CT method. The reactions were performed in duplicate.

### 4.11. Western Blotting Analysis of the Tissues

Tissue samples were mixed with 5× denaturing sample buffer, heated at 95 °C for 10 min, and separated on a Bis-Tris discontinuous 4–12% polyacrylamide gradient gel (NuPAGE; Invitrogen). Proteins were then transferred to PVDF membranes and blocked with PBST supplemented with 5% bovine serum albumin for 60 min. Next, the membranes were incubated with specific primary antibodies (1:1000), including Bax (Santa Cruz Biotechnology), cleaved caspase-3 (CC3) (Cell Signaling Technology), and β-actin (1:10,000; Sigma-Aldrich), in PBST and incubated overnight at 4 °C. The blots were incubated with anti-rabbit HRP-conjugated IgG (1:1000 in PBST; Gene Tex) for 60 min at 25 °C to detect the binding of its corresponding antibody. Immunoreactive bands were detected using an ECL Reagent (Millipore, Billerica, MA, USA). For the quantitative analysis of protein expression, the optical density of protein bands was analyzed using video densitometry (G-box Image System; Syngene, Frederick, MD, USA).

### 4.12. Measurement of Glutathione Peroxidase (GPx) and Malondialdehyde (MDA) Levels

The activity of GPx in aortic tissues and HAECs were determined using Cayman chemicals kits, according to the manufacturer’s instructions. GPx uses reduced glutathione’s ability to reduce hydrogen peroxide. Glutathione reductase is used to regenerate reduced glutathione in the presence of NADPH. The decrease in NADPH absorbance that can be monitored at 340 nm is proportional to the GPx activity in the sample. The level of lipid peroxidation (MDA) in aortic tissue homogenates was evaluated using the thiobarbituric acid (TBA) test and lipid peroxidation (MDA) assay kit (abcam, ab118970). The MDA in the sample reacts with TBA to form the MDA-TBA adduct, which can be easily quantified spectrophotometrically at 532 nm.

### 4.13. Statistical Analysis

All data are presented as a relative frequency for discrete responses and means ± standard deviation for continuous responses. Statistical comparisons were performed using the Student’s *t*-test or one-way analysis of variance (ANOVA), and Scheffé’s method was used for intergroup comparisons. Results with *p* values < 0.05 were considered statistically significant.

## 5. Conclusions

The results indicated that KA inhibited H_2_O_2_-induced endothelial cell damage by directly reducing intracellular ROS generation. These protective effects involved the inhibition of the apoptosis cascade by inhibiting CC3 activation and reducing the IKKβ, IκBα, and NF-κB phosphorylation levels. In vivo, KA reduced lipid accumulation and prevented atherogenic changes in the aortas of *ApoE*^−/−^ mice. Thus, these findings provide novel insights into the preventive role of KA in the development of atherosclerosis and warrant additional studies regarding its potential use as a therapeutic agent.

## Data Availability

The data presented in this study are available in article.
